# Deciphering light transformation in chiral metasurface in real space and time by ultrafast electron microscopy

**DOI:** 10.1038/s41377-025-02163-8

**Published:** 2026-01-14

**Authors:** Ling Tong, Fei Xie, Xiaochen Gao, Yuxuan Chen, Shaozheng Ji, Bin Zhang, Jing Li, Jiangteng Guo, Fang Liu, Cuntao Gao, Min Feng, Wei Wu, Shibin Deng, Yiming Pan, Yunquan Liu, Jingjun Xu, Mengxin Ren, Xuewen Fu

**Affiliations:** 1 The Key Laboratory of Weak-Light Nonlinear Photonics, School of Physics, Nankai University, Tianjin, 300071 China; 2 College of Science, Hebei Agricultural University, Baoding, 071001 China; 3 Department of Electrical Engineering Physical Electronics, Tel Aviv University, Ramat Aviv, 6997801 Israel; 4 State Key Laboratory for Mesoscopic Physics and Collaborative Innovation Center of Quantum Matter, School of Physics, Peking University, Beijing, 100871 China; 5 The TEDA institute of Applied Physics, Nankai University, Tianjin, 300071 China; 6 Academy for Advanced Interdisciplinary Studies, Nankai University, Tianjin, 300071 China; 7 State Key Laboratory of Quantum Functional Materials, School of Physical Science and Technology and Center for Transformative Science, ShanghaiTech University, Shanghai, 200031 China

**Keywords:** Nanophotonics and plasmonics, Metamaterials, Transformation optics, Ultrafast photonics, Transmission electron microscopy

## Abstract

Optical activity in chiral structures, i.e., circular dichroism (CD), has led to significant advances in nanoscale optical manipulation, including chiral metasurfaces, helicoid crystals, and chiral macromolecules. Although the local geometric design of chiral structures fundamentally governs their optical responses, the microscopic origin of CD remains unresolved due to the inability to probe optical chirality generation and local geometry effects with sufficient spatiotemporal resolution. Here, we unveil the light transformation process in a Γ-shaped chiral metasurface by combining far-field ellipticity measurements with direct near-field imaging at nanometer-femtosecond scale using photon-induced near-field electron microscopy (PINEM). By decomposing the near-field distributions into local symmetric and asymmetric components, we define a near-field ellipticity that quantitatively follows the wavelength-dependent far-field ellipticity. Finite-element simulations reveal that an electric dipole at the top-right corner of the Γ-shaped meta-atom dominates the ellipticity, which increases as the dipole contribution grows with wavelength. Crucially, time-resolved PINEM reveals that asymmetric near-fields dissipate faster than the symmetric counterparts by tens to hundreds of femtoseconds, indicating chiral-geometry-dependent energy dissipation pathways. This work provides microscopic insight into light transformation in chiral structures and lays the foundation for advanced chiral photonic device design.

## Abstract (web summary)

Ultrafast electron microscopy imaging of near-field spatiotemporal evolution unveils the microscopic mechanism of light transformation in chiral metasurfaces.

## Graphical abstract

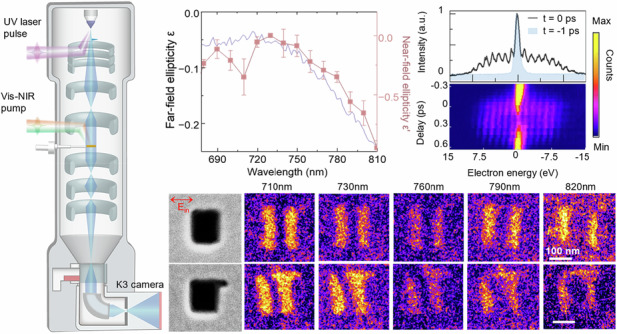

## Introduction

Chirality, the property of an object being non-superimposable onto its mirror image through translations or rotations^1–5^, manifests profound implications across optical devices^6–10^, biosensing^11,12^, and quantum optics^13^. While intrinsic chirality in natural materials is typically weak, recent breakthroughs have demonstrated^1^ that resonant chiral metasurfaces with geometrically engineered asymmetric metamolecules (e.g., L-, G-, and U-shaped architectures^14–17^) can dramatically amplify chiral light-matter interactions. To evaluate the chirality of a metasurface, circular dichroism (CD) that reflects electromagnetic chirality is usually employed, which mainly relies on differential interactions between chiral meta-atoms and electromagnetic fields of opposite handedness and is typically quantified by measuring the absorption differences between left- and right-circularly polarized (LCP and RCP) light within a chiral medium^18^ or by detecting ellipticity changes of transmission light^5,19^. Therefore, understanding the microscopic mechanism of how nanoscale geometric chirality governs these interactions and the light chirality is pivotal for optimizing chiral metasurface design and enabling multifunctional applications.

Recent advances in chiral metasurfaces have primarily focused on far-field characterizations, such as transmission/reflection spectroscopy^20,21^, which typically detect far-field light to quantify the overall CD of chiral structures. However, the spatial resolution of these techniques is fundamentally constrained by the inherent diffraction limit, hindering their ability to resolve the influence of meta-atom geometry on the CD of the chiral metasurfaces. The emerging near-field optical probing techniques, such as cathodoluminescence^22,23^, scanning near-field optical microscopy^24^, photoemission electron microscopy^25^, near-field optical microscopy^26^, and photon-induced near-field electron microscopy (PINEM)^27,28^, have effectively overcome this limitation. Among these, PINEM stands out as a novel approach that integrates pump–probe techniques with transmission electron microscopy (TEM), utilizing ultrafast electron pulses as a probe to characterize time-varying electromagnetic fields at a high spatiotemporal resolution^29–35^. These techniques enable subwavelength-scale investigation of chiral metasurfaces, significantly advancing the characterization of chiral nanostructures by providing detailed insights into near-field distributions. Nevertheless, previous studies have predominantly focused on the near-field behavior of assembled chiral meta-atoms, overlooking the critical contributions of the local geometric features. Consequently, the role of localized near-field asymmetry of chiral meta-atoms in modulating light’s chirality remains elusive, and the correlation between the localized near-field and the CD of chiral metasurfaces remains unexplored.

Here, we address this gap via direct imaging of local near-field distribution in individual meta-atoms within chiral metasurfaces using PINEM at nanometer-femtosecond spatiotemporal resolution within a home-built four-dimensional ultrafast electron microscopy (4D UEM) system. By quantifying the chirality of the near-field distribution at a single meta-atom level, we establish a direct correlation between the local asymmetric near-field distribution and the CD in a typical chiral metasurface composed of a Γ-shaped meta-atom array. Our PINEM imaging reveals that the Γ-shaped meta-atoms in the chiral metasurface exhibit a novel asymmetric near-field distribution, in sharp contrast to the symmetric near-field distribution of rectangular meta-atoms in achiral metasurfaces. The wavelength-dependent ellipticity of the asymmetric near-field closely follows the wavelength-dependent ellipticity angle of the far-field. Finite-element simulations demonstrate that the unique asymmetric near-field distribution in the Γ-shaped meta-atoms is induced by the formation of an electric quadrupole mode under horizontally polarized light illumination, elucidating how the local geometry of the chiral meta-atoms determines the near-field distribution across different wavelengths. Furthermore, within the Γ-shaped meta-atoms, the geometric chirality induced asymmetric near-fields exhibit faster decay dynamics than the symmetric ones aligned along the incident laser polarization, resulting in different free electron–photon coupling processes. These findings establish causality between the microscopic geometry chirality and the macroscopic CD response, providing new insights into the light-matter interactions in chiral structures and paving the way for predictive design of photonic systems for versatile applications, such as biosensing^36^, optical devices^37,38^, and polarization control^39^.

## Results

### Characterization of near-field and far-field chirality of chiral metasurface

**Fig. 1:**
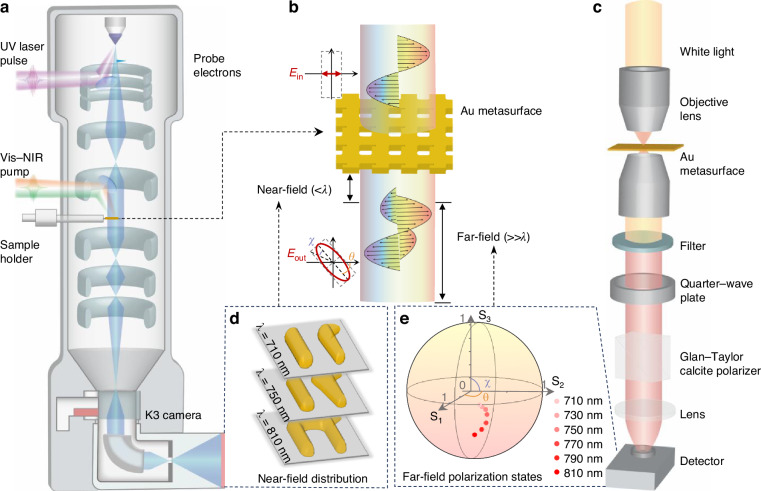
**Near- and far-field measurements of a chiral metasurface**. **a** Schematic diagram of 4D UEM with PINEM imaging capability for near-field measurement with high spatiotemporal resolution. **b** Schematic illustration for the near-field (electromagnetic field within a distance less than *λ*) and far-field (electromagnetic field with a distance much greater than *λ*) evolutions of a chiral metasurface under light illumination. **c** Schematic diagram of a polarimeter used for measuring the polarization azimuth rotation (*θ*) and the ellipticity angle (*χ*) of a chiral metasurface. **d** Schematic of the near-field distributions in a Γ-shaped chiral meta-atom at excitation wavelengths of 710, 750, and 810 nm. **e** Measured polarization states of different output light (710–810 nm) represented on the Poincaré sphere. UV means ultraviolet, and Vis-NIR means visible-near infrared

As electromagnetic waves transition from the near-field to the far-field in chiral structures, they evolve from a strong localized non-radiative regime to a propagating radiative field^40^, as schematically illustrated in Fig. 1b. To investigate the relationship between the near-field distribution and the far-field chirality, we performed measurements on both far-field and near-field characteristics of the same chiral metasurface sample. The near-field measurements were conducted using PINEM imaging in a home-built 4D UEM based on a field-emitter transmission electron microscope (TEM) (see Fig. 1a). The mechanisms of 4D UEM and PINEM have been described elsewhere^28,41–46^. Briefly, a femtosecond (fs) laser beam is split into two components: one is used to pump a noncollinear optical parametric amplifier (NOPA) to generate wavelength-tunable 30 fs pulse (680–830 nm) for sample excitation, while the other one is used to pump another NOPA to generate 30 fs UV (300 nm) pulse for producing fs electron pulses as probe. The time delay between the pump and probe pulses is controlled by an optical delay stage. With an electron energy filter in the 4D UEM, PINEM imaging can be performed in both TEM and scanning transmission electron microscope (STEM) modes, enabling direct imaging of optical near-field distribution in real space and time (see Fig. 1d, “Materials and methods”, Supplementary Note 1 and Fig. S1). For the far-field characterization, we utilized a high-precision polarimeter consisting of a rotating superachromatic quarter-wave plate (WP) and a Glan–Taylor (GT) calcite polarizer^19^, as schematically shown in Fig. 1c. When horizontally polarized light passes perpendicularly through the chiral metasurface, the variations in ellipticity angle (*χ*) and polarization azimuth rotation (*θ*) of the transmitted light are recorded by the polarimeter (see Fig. 1e, “Materials and methods”). Combining these two techniques, we can establish a direct correlation between the local near-field distribution and the far-field polarization characteristics (Fig. 1d, e).

**Fig. 2:**
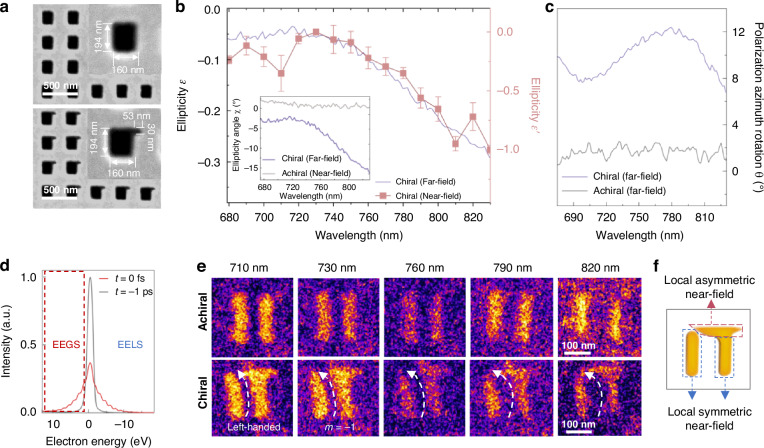
**Far-field and near-field characteristics of achiral and chiral metasurfaces**. **a** Typical scanning electron microscopy images of an achiral (top panel) and chiral (bottom panel) Au metasurfaces. **b** Wavelength-dependent changes of ellipticity *ε* of the transmitted light for the chiral metasurfaces under horizontally polarized light illumination (680–830 nm). Inset: Ellipticity angle (*χ*) for chiral and achiral metasurfaces. Error bars represent standard deviations from three independent measurements. **c** Wavelength-dependent changes of polarization azimuth rotation *θ* of the transmitted light for the chiral and achiral metasurfaces, respectively, under horizontally polarized light illumination (680–830 nm). **d** Electron energy spectra (including energy gain (EEGS) and loss (EELS)) at *t* = 0 fs and *t* = −1 ps obtained from a single meta-atom of the chiral metasurface under 830 nm fs laser excitation. **e** PINEM imaging results of the near-field distributions (*t* = 0 fs) in a single meta-atom of the achiral (top panel) and chiral (bottom panel) metasurfaces under fs laser excitation with different wavelengths. The PINEM images were obtained by selecting electrons at the energy gain side, as indicated by the red-dashed rectangle in (**d**). **f** Schematic diagram illustrating the local symmetric and asymmetric near-field components within a chiral meta-atom

To better establish a direct correlation between the local near-field distribution and the CD of the chiral metasurface, we systematically investigated the near-field and far-field characteristics of both chiral (composed of chiral Γ-shaped meta-atoms) and achiral (composed of achiral rectangular meta-atoms) metasurfaces with identical dimensions for comparison (Fig. 2a). The samples were fabricated via focused ion beam (FIB) milling through an 80-nm-thick gold film deposited on a 30-nm-thick Si_3_N_4_ substrate. The metamolecules of the chiral metasurface were intentionally designed in a “Γ” shape, with the asymmetry stemming exclusively from a distinct modification in the top-right corner compared to the achiral counterpart (Fig. 2a). Both metasurfaces consisted of 64×64 arrays of metamolecules with identical periodicity on the same film (Fig. S2). It has been demonstrated that such a Γ-shaped chiral metasurface transforms horizontally polarized light into elliptically polarized light^19,47^, as schematically shown in Fig. S3. As shown in the inset of Fig. 2b, the far-field ellipticity angle (*χ*) of this chiral metasurface is below −3° and displays a continuous decrease to −16° from 730 to 830 nm, indicating a remarkable wavelength-dependent left-handed polarization change of the transmitted light. In contrast, the ellipticity angle of the achiral metasurface remains around zero and exhibits negligible variation in the wavelength range of 680–830 nm, implying that the transmitted light undergoes no polarization change. Similarly, the polarization azimuth rotation angle (*θ*) of the chiral metasurface exhibits a strong wavelength dependence, reaching around 10°, while that of the achiral metasurface shows no visible wavelength dependence, remaining around 0° (Fig. 2c). These results unambiguously demonstrate that the chiral metasurface can significantly alter the ellipticity and polarization state of the transmitted light, implying selective absorption of a specific circular polarization component of the incident light. For the studied chiral metasurface in this work, it has a larger absorption of the RCP component than the LCP component under horizontally polarized light illumination (Fig. 2b).

We then turn to near-field characterization of the metasurfaces by PINEM imaging. Figure 2d shows two electron spectra of a single meta-atom in the chiral metasurface under fs laser excitation at 830 nm, where the spectrum at *t* = −1 ps represents the probe electrons without interaction with the near-field, while the spectrum at *t* = 0 fs corresponds to the electron packet at its maximum interaction with the near-field. Electrons that gained energy at *t* = 0 fs spectrum were utilized for PINEM imaging to map the near-field distribution. For the rectangular meta-atom in achiral metasurface, its near-field distribution is symmetric and aligns perpendicular to the polarization direction of the incident light, with negligible wavelength dependence in the near-field distribution (top panel of Figs. 2e and S4a). In contrast, for the Γ-shaped meta-atom in the chiral metasurface, the structural chirality induces near-field distortion, giving rise to a component deviating from the original polarization direction. This results in a pronounced asymmetric near-field distribution nearly parallel to the incident polarization at the top region (Figs. 2e and S4b), which we term the local asymmetric near-field. Notably, similar to the pronounced wavelength dependence observed in the far-field ellipticity, the asymmetric near-field distribution also demonstrates significant wavelength sensitivity, particularly within the 730–830 nm wavelength range. Specifically, the local near-field distribution extends from right to left in the top region as the wavelength increases. This observation suggests a strong correlation between the near-field and far-field behaviors of the chiral metasurface.

The chirality of the transmitted light is determined by both its ellipticity angle *χ* and polarization azimuth angle *θ* (Fig. 1e). Since the *θ* induced by the chiral metasurface is relatively small, approximately 8–10° in Fig. 2c, distinguishing its effect on the near-field distribution is challenging (Fig. S5). Moreover, the azimuth rotation primarily corresponds to a global rotation of the main optical axis, unlike the local asymmetric extension observed in the near-field. Therefore, the significant change of the local near-field distribution within the wavelength range between 730–830 nm is primarily attributed to the ellipticity variation. To quantitatively analyze their correlation, we introduce an ellipticity $({\varepsilon}^{{\prime}})$ for the near-field, defined as ${\varepsilon }^{{\prime} }={I}_{{as}}/{I}_{s}\times m$, to quantify its chirality. Here, ${I}_{{as}}$ represents the integral intensity of the local asymmetric near-fields (indicated by the red-dashed box in Fig. 2f), and ${I}_{s}$ is the integral intensity of the local symmetric near-fields (indicated by the blue dashed boxes in Fig. 2f). The parameter *m* characterizes the overall distribution rotation direction of the near-field, where *m* = −1 indicating a counterclockwise rotation and *m* = 1 representing a clockwise rotation. The counterclockwise and clockwise rotations of the near-field are defined by referring to the distortion directions (indicated by the dashed arrows in Fig. S6c) of the near-field distribution in the achiral rectangular meta-atom under elliptically polarized light illumination with left-handed and right-handed chirality, respectively. The bottom panel of Fig. 2e shows that under the horizontally polarized light illumination, the near-field of the Γ-shaped meta-atom exhibits a counterclockwise rotation, i.e., *m* = −1. The detailed definition and derivation of the near-field ellipticity $({\varepsilon}^{{\prime}})$ are provided in Supplementary note 2. As shown in Fig. 2b, the calculated $\varepsilon^{\prime}$ of the near-field aligns well with the far-field ellipticity $\varepsilon$ over the wavelength range of 680–830 nm, indicating that the emergence of asymmetric near-fields in the chiral metasurface directly correlates with the CD of the far-field light. This correlation between near-field ellipticity $({\varepsilon}^{{\prime}})$ and far-field ellipticity (*ε*) was further confirmed in a 100-nm-thick gold Γ-shaped metasurface (Fig. S9) and a 100-nm-thick gold L-shaped metasurface (Fig. S10), verifying the universality of the relationship across different geometric configurations. The wavelength-dependent ellipticity of the asymmetric near-field retrieved from Figs. 2e and S4b directly mirrors the far-field CD (Fig. 2b), confirming that the macroscopic optical activity originates from the localized geometric asymmetry. Notice that all analyzed meta-atoms were selected from the central region of the 64×64 metasurface arrays to minimize edge effects.

### Origin of the asymmetric near-field and mechanism behind the ellipticity change for the chiral metasurface

**Fig. 3:**
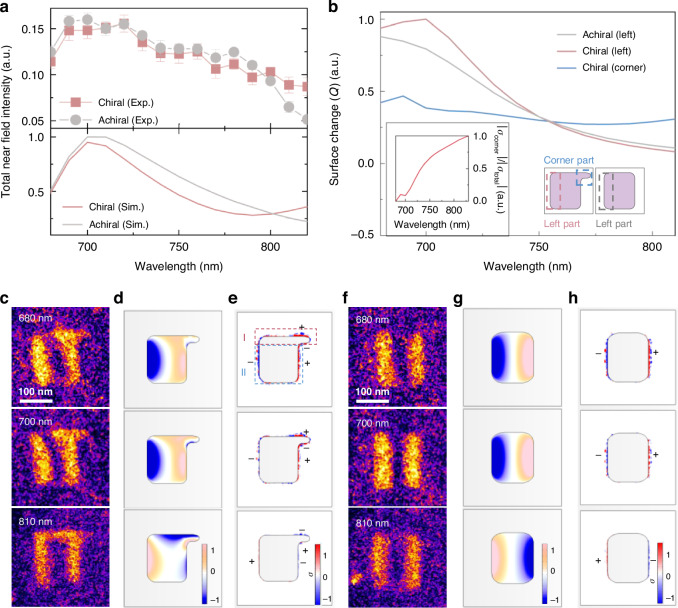
Finite-element electromagnetic wave simulation for the chiral and achiral metasurfaces. **a** Top: Integral near-field intensity of the chiral and achiral metasurfaces measured under horizontally polarized light illumination with wavelength spanning from 680 to 830 nm. Error bars represent standard deviations from five independent measurements. Bottom: Corresponding simulated integral electric field intensity of the chiral and achiral metasurfaces. **b** Wavelength-dependent surface charge variations in the local regions of the chiral and achiral meta-atoms under horizontal polarization excitation. Left inset: Wavelength-dependent ratio of corner surface charge to the total surface charge in the chiral structure. Right inset: Schematic of the selected regions in the meta-atoms. **c**, **f** Measured near-field distributions of chiral and achiral meta-atoms at three typical wavelengths of 680 nm, 700 nm, and 810 nm. **d**, **g** Simulated electric field (*E*_*z*_) distributions for the chiral and achiral meta-atoms, respectively. **e**, **h** Corresponding simulated charge distributions for the chiral and achiral meta-atoms, respectively. The charge distributions (*σ*, surface charge density, color maps) were retrieved from the top surface of the metasurface

To elucidate the origin of the asymmetric near-field distribution and reveal the underlying mechanism for the observed changes in light ellipticity, we performed finite-element electromagnetic simulations based on the actual geometry of the metasurfaces (see “Materials and methods”). The simulated transmission ratio and ellipticity angle *χ* match well with the experimental results (Fig. S11), confirming the validity of our simulations. On this basis, we further investigated the main factors that influence the near-field ellipticity in the chiral metasurface. As shown in Fig. 3a, the near-field intensity of the chiral and achiral metasurfaces exhibits a pronounced plasmonic resonance peak in the wavelength range of 680–720 nm, while their ellipticity degree in this wavelength range remains relatively small (Fig. 2b), suggesting that these strong near-fields have little effect on the ellipticity. In contrast, for wavelengths between 790 and 830 nm, the chiral Γ-shaped meta-atoms exhibit stronger near-fields than the achiral rectangular meta-atoms (Fig. 3a), particularly due to the enhanced asymmetric near-field at the top of the Γ-shaped meta-atom. Our results demonstrate that the near-field ellipticity is determined by the relative contribution of the asymmetric component, rather than the overall near-field intensity. Analysis of the surface charge (Fig. 3b) reveals that while the left-side integrated charge shows a similar decreasing trend beyond 700 nm for both chiral and achiral structures, the corner surface charge in the chiral meta-atom exhibits a subtle increase with the wavelength. Consequently, the proportion of corner surface charge relative to the total surface charge gradually grows (inset of Fig. 3b), leading to an increasingly dominant influence on the overall near-field distribution. Thus, when the plasmonic resonance peak and the corner-localized resonance peak are spectrally well separated, the near-field ellipticity of the chiral metasurface is primarily governed by the asymmetric feature at the top-right corner of the Γ-shaped meta-atom.

We further analyzed the near-field distributions (experimental results in Fig. 3c, f; simulated results in Fig. 3d, g) and the corresponding charge distributions (Fig. 3e, h) in the chiral and achiral meta-atoms under horizontally polarized light illumination at three typical wavelengths: 680 nm, 700 nm, and 810 nm. For both chiral and achiral metasurfaces, the simulated near-field distributions agree well with the experimental results, providing a solid basis for analyzing their microscopic charge distributions. For the rectangular meta-atom of the achiral metasurface, the charge distribution exhibits a simple dipole mode (Fig. 3h), which shows little variation with the illumination wavelength. In contrast, the Γ-shaped meta-atom of the chiral metasurface exhibits an asymmetric quadrupole electric field distribution due to the emergence of a small dipole distribution at the top-right corner. This quadrupole electric field distribution can be divided into two parts: (1) the region “I,” indicated by the red-dashed rectangle at the top of the Γ-shaped meta-atom (top panel of Fig. 3e), which is strongly influenced by the asymmetric top-right corner; and (2) region “II,” outlined by the blue dashed rectangle at the bottom of the Γ-shaped meta-atom (top panel of Fig. 3e), which resembles the dipole distribution in the rectangular meta-atom of the achiral metasurface. In the 680–720 nm wavelength range, region “II” dominates the light absorption, causing the near-field of the chiral Γ-shaped meta-atom to exhibit a dipole-like distribution (Fig. 3c, f). As the wavelength increases to 730–830 nm, the relative contribution of the corner dipole rises (inset of Fig. 3b), amplifying the field intensity in region “I” and resulting in a distinct “n”-shaped near-field distribution at 810 nm (bottom panel of Fig. 3c).

### Temporal behavior of the asymmetric near-field distribution in the chiral metasurface

**Fig. 4:**
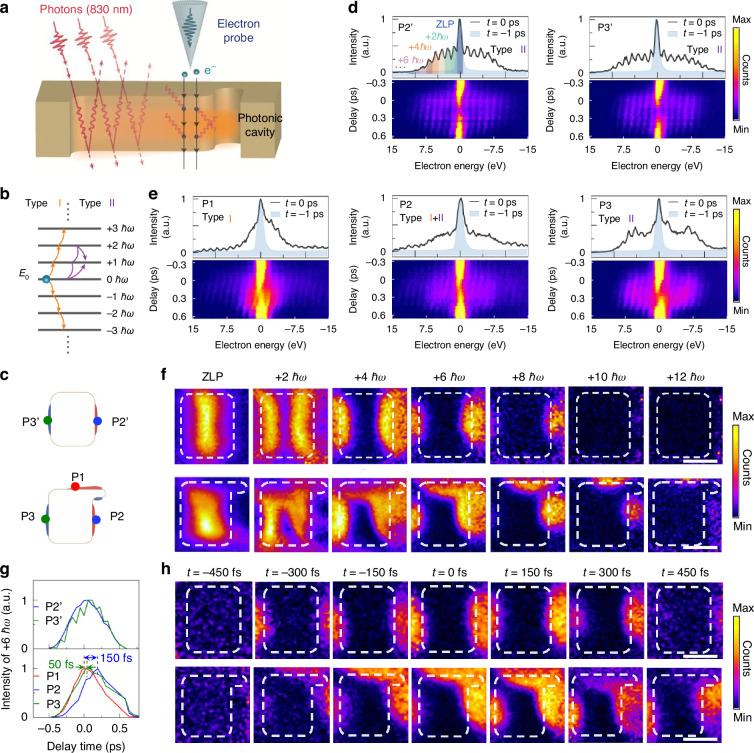
Probing interaction of free electrons with localized near-fields in a metal photonic cavity by PINEM. **a** Schematic of PINEM measurement under STEM mode, in which a fs electron probe scans over the localized near-fields in a chiral meta-atom under a fs laser pulse excitation. **b** Energy level diagram of ladder states with spacing *ħω* relative to the initial state at *E*_*0*_. Arrows indicate sequential multistate population transfer (Type I) and interfering quantum paths (Type II), leading to multilevel Rabi oscillations. **c** Schematic representation of electric dipole and electric quadrupole models in the achiral and chiral meta-atoms. **d**, **e** Top: Electron energy spectroscopy data showing multiple photon absorption and emission collected at P1 to P3 (chiral) and P2′ to P3′ (achiral) positions indicated in (**c**). Areas of different colors in the PINEM spectrum of P2′ position indicate electron energy gained by different numbers of photons. Bottom: Corresponding time-resolved difference electron energy spectroscopy map. **f** Energy gain images obtained at time zero with the selected energy states of +0 *ħω*, +2 *ħω*, +4 *ħω*, ……, and +12 *ħω* for the achiral (top panel) and chiral (bottom panel) meta-atoms. **g** Probabilities of the electron–photon interaction intensity at +6 *ħω* of different localized near-fields versus delay time. **h** Time-dependent PINEM energy-gain images at +6 *ħω* for the achiral (top panel) and chiral (bottom panel) meta-atoms (see **d**, **e** for the energy gain spectra). White dashed lines indicate the meta-atom boundaries, where the inner area represents the vacuum region and the outer area is the Au film. Scale bar: 100 nm

Previous studies on near-field dynamics of metasurfaces have mainly focused on ensemble achiral structures^48^, leaving the critical local interactions, especially in chiral metasurfaces, largely unexplored. Here, we employed PINEM measurements in STEM mode to investigate the interaction between free electrons and localized near-fields with high spatiotemporal resolution, as schematically illustrated in Fig. 4a. Typically, the interaction between free electrons and near-field generates electron energy sidebands on both sides of the electron energy spectrum separated from the initial energy *E*_0_ by integer multiples of the photon energy *nħω* (*n* is an integer number, indicating the photon order)^41,42^. Each sideband corresponds to the absorption (energy gain) or emission (energy loss) of an integer number of the incident photons, and the interaction strength is encoded in the number of populated sidebands and their individual amplitudes. In this scenario, each electron is dressed into an infinite quantum energy ladder equally spaced by the incident photon energy *ħω* (Fig. 4b). Generally, there are two types of interaction processes between the free electrons and the localized near-fields, namely, type I and type II processes (denoted in Fig. 4b)^42^. Type I process is characterized by sequential multilevel excitations, leading to a partial reduction in the initial electronic state population and spectral broadening, with the sideband intensity distribution monotonously decaying toward higher photon orders; while type II process is dominated by multipath interference, resulting in multilevel Rabi oscillations^49^. We found that the spectrum collected from the P1 position of the chiral meta-atom (indicated in the bottom panel of Fig. 4c) shows the typical feature of the type I interaction process (Fig. 4e**)**, whereas the spectra collected from the P2′ and P3′ positions (indicated in the top panel of Fig. 4c) of the achiral meta-atom (Fig. 4d), as well as from the P3 position (indicated in the bottom panel of Fig. 4c) of the chiral meta-atom (right panel of Fig. 4e) exhibit the type II interaction process. More interestingly, the optical phase modulation of the free electrons by the near-field at the P2 position (indicated in the bottom panel of Fig. 4c) exhibits a combination of type I and II interaction behaviors. The distinct interaction behaviors among the P1, P2, and P3 positions of the chiral meta-atom indicate that, unlike the symmetric structures (e.g., photonic crystals^32^ and achiral meta-atoms), the interaction between free electrons and near-field within the chiral meta-atom exhibits gigantic spatial diversity due to the asymmetric local geometry. Note that this intriguing phenomenon is also related to the distinct dissipation dynamics of the localized near-fields at different positions, as further discussed in the following.

Figure 4f displays the PINEM energy gain images obtained at time zero by selecting electrons at energy states of 0 *ħω*, +2 *ħω*, +4 *ħω*, ……, and +12 *ħω* for the achiral (top panel) and chiral (bottom panel) meta-atoms at 830 nm excitation with a fluence of 14.6 mJ cm^−2^. The chiral meta-atom supports interactions up to +12 photons (+12 *ħω*), exceeding the achiral structure’s limit of +10 photons (+10 *ħω*), implying that the chiral nanostructure possesses a broader spectrum and enhanced photon capacity.

It has been reported that beyond phase-matching conditions, the electron–photon coupling strength also depends on the decay process of the near-fields^32,50^. For example, in photonic cavities, high-*Q* modes sustain longer interaction times than low-*Q* modes, with the maximal interaction time-point being delayed by ~200 fs due to the time-dependent accumulation of optical energy within the cavity^32^. Figure 4d, e (bottom panels) show the time-dependent evolution of the electron energy spectra. Integration of specific energy-gain peaks allows extraction of the maximal interaction time (corresponding to the peak position on the time axis) and the temporal convolution (full width at half maximum (FWHM)) between the electron pulse and the optical near-field (Materials and methods). Since the near-field changes induced by absorbing higher-order photons are more sensitive to capturing the maximum interaction time-point compared to multiple orders, we further collected PINEM energy gain images by selecting electrons with +6 *ħω* energy gain to further reveal the specific maximal interaction time points of the three positions (Fig. 4h). As shown by the time-dependent probabilities of interaction intensity in Fig. 4g, the maximum interaction times of the symmetric near-fields at the P2 and P3 positions exhibit a time shifts of about +50 fs and +150 fs, respectively, relative to that of the asymmetric near-field at P1 position. This temporal disparity indicates a faster dissipation of the asymmetric near-field at the P1 position than the symmetric fields at the P2 and P3 positions. Under vertically polarized 830 nm fs laser excitation, the asymmetric near-fields deviating from the laser’s original polarization also dissipate faster than the symmetric ones (see Fig. S12). The faster dissipation of the localized asymmetric near-fields, primarily governed by structural chirality in the chiral meta-atom, results in the dramatic spatial diversity in the interaction process between free electrons and localized near-fields (type I or type II process), providing significant new insights into the nanoscale ultrafast light manipulation with chiral metasurfaces.

## Discussion

In this study, we have systematically investigated the light transformation dynamics in chiral metasurfaces at the femtosecond–nanometer scale by PINEM imaging. Under horizontally polarized light illumination within the 730–830 nm wavelength range, the top-right corner of the Γ-shaped meta-atom in the chiral metasurface introduces a localized asymmetric near-field, exhibiting a spatial distribution that gradually extends from right to left across the top region with increasing excitation wavelength. Through quantitative analysis, we established a direct correlation between the near-field ellipticity $({\varepsilon}^{{\prime}})$ and the far-field ellipticity (*ε*), revealing that the macroscopic CD originates from the asymmetric near-field induced by the geometric asymmetry of individual meta-atoms. Finite-element simulations further demonstrate that the wavelength-dependent variation in the ellipticity is primarily governed by the increased contribution of the electric dipole at the top-right corner. Crucially, time-resolved PINEM measurements revealed that the asymmetric near-fields exhibit faster decay dynamics than the symmetric counterparts by 25–150 fs, indicating remarkable chirality-dependent energy dissipation pathways. This faster dissipation, attributed to the structural asymmetry of the Γ-shaped meta-atoms, results in distinct free-electron–near-field interaction processes. This work not only elucidates the microscopic mechanism of chiral light-matter interactions but also establishes a causal link between nanoscale geometry and macroscopic optical response, which provides a unified framework for the rational design of chiral photonic devices with broad applications in chiral light sources^51^, ultrafast polarization control^52–54^, and advanced biosensing^55^.

## Materials and methods

### TEM specimen preparation and characterization

The metasurfaces were fabricated using FIB milling through an 80/100-nm-thick gold film deposited on a 30 nm thick Si_3_N_4_ substrate (Ted Pella, Inc.). The detailed geometry parameters of a metamolecule are shown in the inset of Fig. 2a. Both chiral and achiral metasurfaces were constructed as 64 × 64 arrays of metamolecules, with a periodicity of 350 nm across all samples. The meta-atoms were fabricated in matrix etched mode with a current of 7.7 pA and a voltage of 30 kV in an FIB system (Helios 5 UC Dual Beam, FEI Inc.).

### PINEM experiment

The measurements were carried out using an ultrafast transmission electron microscope (UTEM) based on a Schottky field-emission TEM (200 kV Talos F200i, FEI Ltd). The UTEM was operated in a pump–probe configuration, driven by a 100 W, 1030 nm, ~250 fs laser (Spectra Physics) running at 200 kHz, whose output was split into two paths by a beam splitter. These two beams were used to drive two noncollinear optical parametric amplifiers (NOPA2H and NOPA3H) separately. NOPA3H delivered a 300 nm laser beam of short pulse duration (30 fs) and eventually generated the photo-electron probe. NOPA2H delivered another beam with a tunable wavelength (660–830 nm, 30 fs pulse duration) to pump the sample. Inside the TEM column, an additional aluminum mirror deflected the pump pulse so that it impinged on the specimen from above at a small angle of ~4° to the *z*-axis. A pump spot of ~60 μm in diameter and a fluence of 7.3 mJ cm^−2^ was formed on the specimen, serving as the optical excitation of the near field. A motorized delay line controlled the temporal offset between the optical pump pulse and the electron probe. To analyze the post-interaction electron energy spectrum, a post-column spectrometer (Gatan) coupled to a retractable K3 Summit direct electron-detection camera was employed. This system also enabled high signal-to-noise ratio energy-filtered TEM (EFTEM) imaging for real-space mapping.

### Far-field experiment

The experimental setup employed a broadband supercontinuum laser source (YSL Photonics, 6W-SC-Pro), whose output light was polarized along the *x*-direction using a GT calcite polarizer. For optical configuration, illumination was implemented through a 10× microscope objective (0.3 N.A.) while light collection utilized a 50× objective (0.8 N.A.). Subsequent polarization analysis of chiral metasurface emissions was performed using a home-built polarimetric system consisting of a rotating superachromatic quarter-WP, GT polarizer, and a fiber-coupled spectrometer.

### Data analysis

The interaction probabilities were determined from time-resolved electron energy spectra acquired using PINEM. For each delay time *t* between the electron and laser pulses, the probability of an electron participating in an *n*-photon interaction process was determined by integrating the signal over the energy window of the corresponding energy gain sideband (e.g., +*n*ℏ*ω*) after subtracting the background.

To resolve the temporal dynamics in Fig. 4g, the delay time was scanned sequentially. The integrated intensity of a selected sideband (+6ℏ*ω*) was used to construct the interaction probability as a function of delay, denoted as *Pn*(*t*). The resulting temporal trace was fitted with a Gaussian function to extract the effective interaction duration (FWHM) and the time of peak interaction strength.

### Numerical simulation

The simulations were performed using the finite-element method (Wave Optics module of COMSOL Multiphysics software) for the results shown in Fig. 3. The Au metasurface was modeled as a square film with a side length of 350 nm. The thickness of the top Au film is 80 nm, while the bottom Si_3_N_4_ film is 30 nm. The geometric parameters of the meta-atom are shown in Fig. 2a. The refractive index of the Si_3_N_4_ substrate was set to be a constant of 2.09. Periodic boundary conditions were applied along the *x* and *y* directions, while the wave interacted with the sample along the *z*-axis. Unphysical radiation reflection from external boundaries was absorbed by the addition of perfectly matched layer subdomains.

The excitation was modeled as a linearly polarized plane wave with a wavelength range from 680 nm to 830 nm. Based on the calculated data, PINEM images (Fig. 3d, g) were simulated using the formula $\frac{1}{\omega L}{\int }_{-L/2}^{L/2}dz{E}_{z}\left(x,y,z\right){e}^{-i\omega z/v}$, as detailed in Supplementary Note 1. Here, *L* is the effective height of the near-field area along the *z*-axis, *v* is the electron’s group velocity, and *ω* is the frequency of the laser.

## Supplementary information

Supplementary Materials

## Data Availability

The data that support the findings of this study are available from the corresponding authors upon reasonable request.
