# Supplementary material for: Deciphering light transformation in chiral metasurface in real space and time by ultrafast electron microscopy
Source: Light Sci Appl. 2026 Jan 14;15:70. doi: 10.1038/s41377-025-02163-8 (PMC12800185; doi:10.1038/s41377-025-02163-8)
Supplement: Supplementary file 1 — Supplementary Materials [file 41377_2025_2163_MOESM1_ESM.docx]

Supplementary Information for

Deciphering light transformation in chiral metasurface in real space and time by ultrafast electron microscopy

Ling Tong^1,†^, Fei Xie^2,†^, Xiaochen Gao^1^, Yuxuan Chen^1^, Shaozheng Ji^1^, Bin Zhang^3^, Jing Li^4^, Jiangteng Guo^1^, Fang Liu^1^, Cuntao Gao^1^, Min Feng^1^, Wei Wu^1^, Shibin Deng^1^, Yiming Pan^5^, Yunquan Liu^4,*^, Jingjun Xu^1,*^, Mengxin Ren^1,*^ and Xuewen Fu^1,6,*^

**Affiliations:**

^1^The Key Laboratory of Weak-Light Nonlinear Photonics, School of Physics, Nankai University, Tianjin 300071, China

^2^College of Science, Hebei Agricultural University, Baoding 071001, China

^3^Department of Electrical Engineering Physical Electronics, Tel Aviv University, Ramat Aviv 6997801, Israel

^4^State Key Laboratory for Mesoscopic Physics and Collaborative Innovation Center of Quantum Matter, School of Physics, Peking University, Beijing 100871, China

^5^School of Physical Science and Technology, ShanghaiTech University, Shanghai 201210, China

^6^Academy for Advanced Interdisciplinary Studies, Nankai University, Tianjin 300071, China

^†^These authors contributed equally to this work.

^*^Corresponding author. Email:

[yunquan.liu@pku.edu.cn](mailto:yunquan.liu@pku.edu.cn); [jjxu@nankai.edu.cn](mailto:jjxu@nankai.edu.cn); [ren_mengxin@nankai.edu.cn](mailto:ren_mengxin@nankai.edu.cn); [xwfu@nankai.edu.cn](mailto:xwfu@nankai.edu.cn)

**This document includes:**

**Supplementary Notes**

**S1.** Electron-filtered transmission electron microscopy (EFTEM) and ultrafast spectral imaging (USI)

**S2.** Definition and validation of the near-field ellipticity

**Supplementary Figures**

Figures 1-12 and captions

**Supplementary note 1. Electron-filtered transmission electron microscopy (EFTEM) and ultrafast spectral imaging (USI)**

In four-dimensional electron microscopy (4D EM), near-field imaging can be performed with two modes: a transmission electron microscopy (TEM) mode, which corresponds to energy-filtered transmission electron microscopy (EFTEM), and a scanning transmission electron microscopy (STEM) mode, corresponding to ultrafast spectral imaging (USI). EFTEM (**Fig. S1b**) is a widely used near-field imaging method capable of detecting features from about 15 nm to the micrometers scale by adjusting the size of the femtosecond (fs) electron beam in ultrafast transmission mode. For features smaller than 15 nm, USI enables high-resolution near-field characterization by scanning the sample pixel by pixel with a focused fs electron beam. The integration of EFTEM and USI provides high signal-to-noise ratio imaging over the nano- to micrometer scale, allowing in-depth investigation of the near-field dynamics of chiral metasurfaces.

Under laser excitation, the interaction between free electrons and laser pulses is mediated by the near-field and satisfies energy–momentum conservation. This interaction causes electrons to gain or lose integer multiples of the photon energy *ħω* (*ħ*, reduced Planck constant; *ω*, frequency)^1, 2^, resulting in sideband peaks that appear symmetrically on both sides of the zero-loss peak (**Fig. S1c** and **Fig. 2d**). These sidebands reflect the electric equipotential regions within the confined near-field integral. The probabilities of electrons occupying different energy states $\left. |n \right\rangle$, shown in **Fig. 2d**, can be determined by integrating the near-field at specific position of (*x, y*) ^1, 3-6^ as

$$g\left( x,y \right)=\frac{e}{\hbar\omega}\int_{-L/2}^{L/2} dzE_{z}\left( x,y,z \right)e^{-i\omega z/v}$$

Here, $g$ represents the spatial integral of the *z*-component of the near-field, $L$ is the effective height of the near-field area along the *z*-axis, *e* is the elementary charge, $v$ is the electron’s group velocity, and $\omega$ is the frequency of the laser. Correspondingly, the probability of electrons at $\left. |n \right\rangle$ is given by

$$P_{n}\left( x,y \right)=J_{n}^{2}\left( 2\left| g\left( x,y \right) \right| \right)$$

where $J_{n}$is the Bessel function of the first kind of order $n$. Energy-filtered images of specific quantum states reveal the spatial distribution of the plasmon field and its spectral characteristics. By selecting the electrons of different energy states ($n$ = 1, 2, 3, etc.), as indexed in **Fig. 4d**, one can quantitatively image individual photon quantum states. The near-field distribution of the Au meta-atom (**Fig. S1c**) shows the characteristics of the mixed photon state by selecting all electrons with gain energy (indicated by the red dotted line) at time zero. Therefore, by varying the time delay between the pump and probe pulses, it is possible to study the near-field dynamics in the Au metasurface on an ultrafast timescale.

**
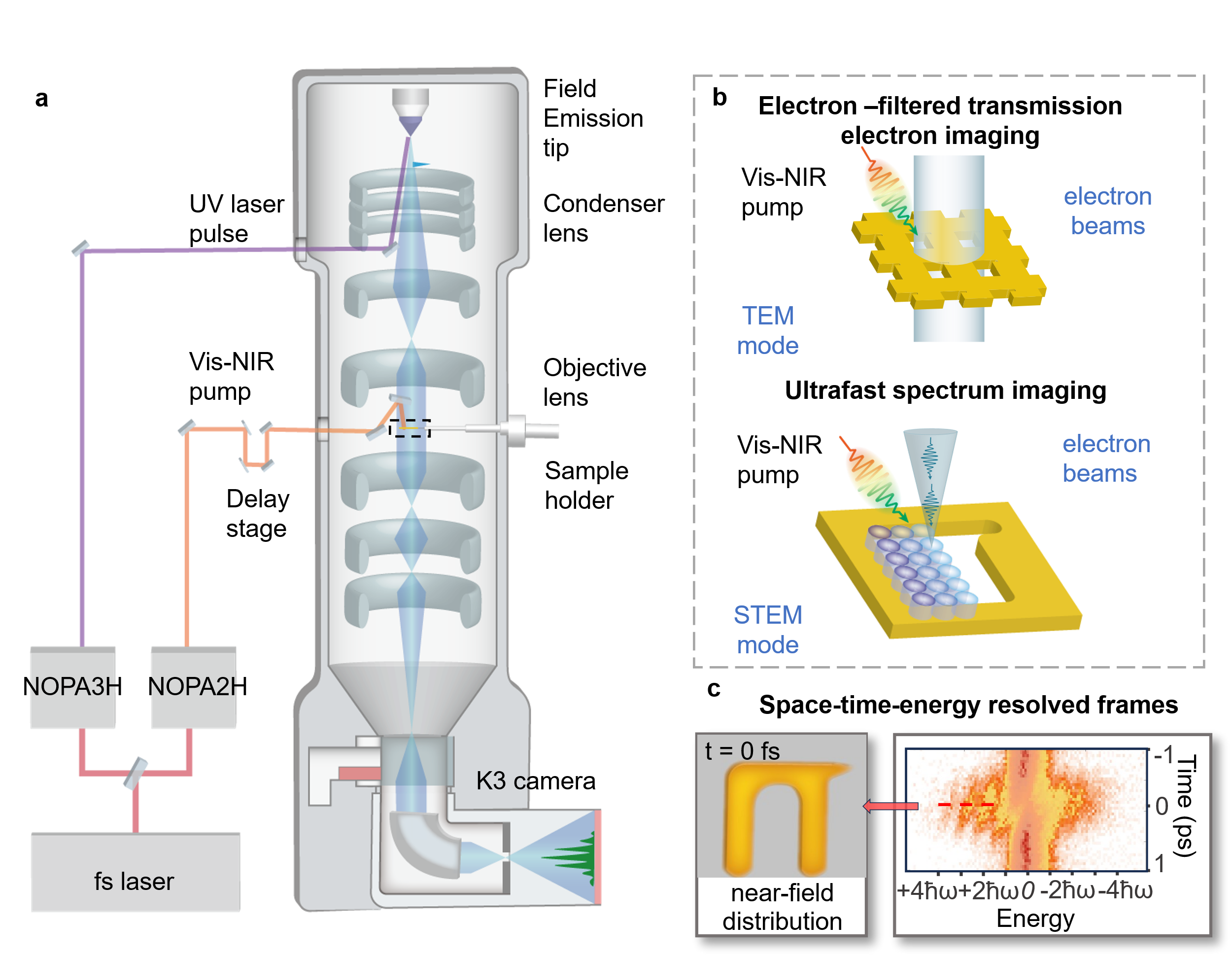
**

**Fig. S1** **Near-field imaging of** **Au chiral** **metasurfaces with photon-induced near-field electron microscopy (PINEM)**. **a**, Schematic diagrams illustrating the optical and electrical constituents of the 4D EM system. Femtosecond laser pulses (300 nm, ~30 fs) excite the cathode inside the TEM to generate fs electron pulses for imaging. Simultaneously, wavelength-tunable fs pulses (from 680 to 830 nm) illuminate the specimen. The time delay between the pump and probe pulses is controlled by a motorized optical delay stage. **b**, An enlarged view of the black dashed box in **a**, elucidating two imaging methodologies employed for near-field characterization: EFTEM mode (top) and USI mode (bottom). **c**, Near-field distributions (left) and time-dependent electron energy spectrum (right) are acquired to map the space-time-energy coordinates.


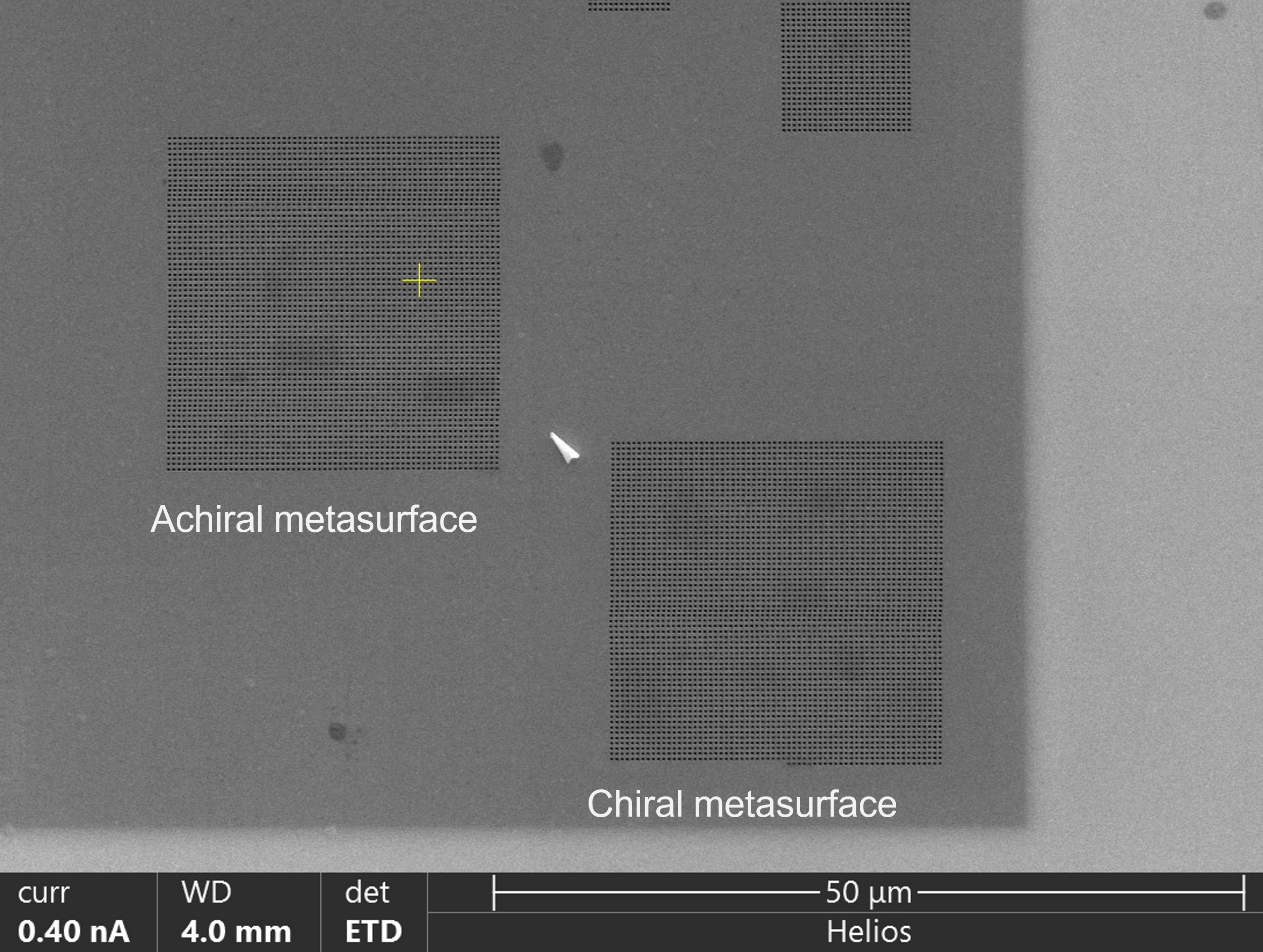


**Fig. S2** Scanning electron microscopy (SEM) images of the achiral and chiral Au metasurfaces.


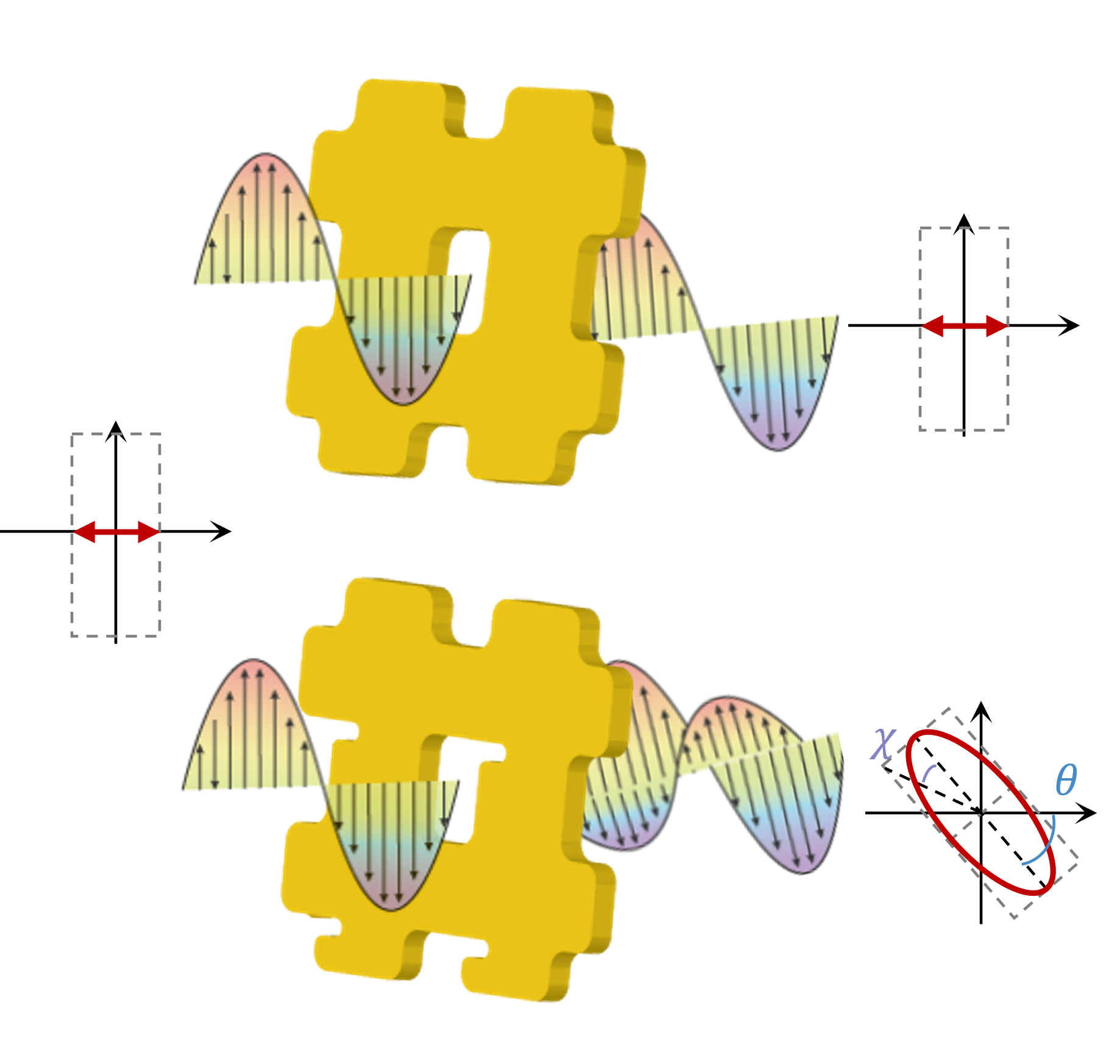


**Fig. S3** Chiral metasurfaces transform a linearly polarized wave into elliptical polarization, a feature not observed in achiral metasurfaces. Changes in polarization states are determined by the polarization azimuth rotation (*θ*) and the ellipticity angle (*χ*).

**
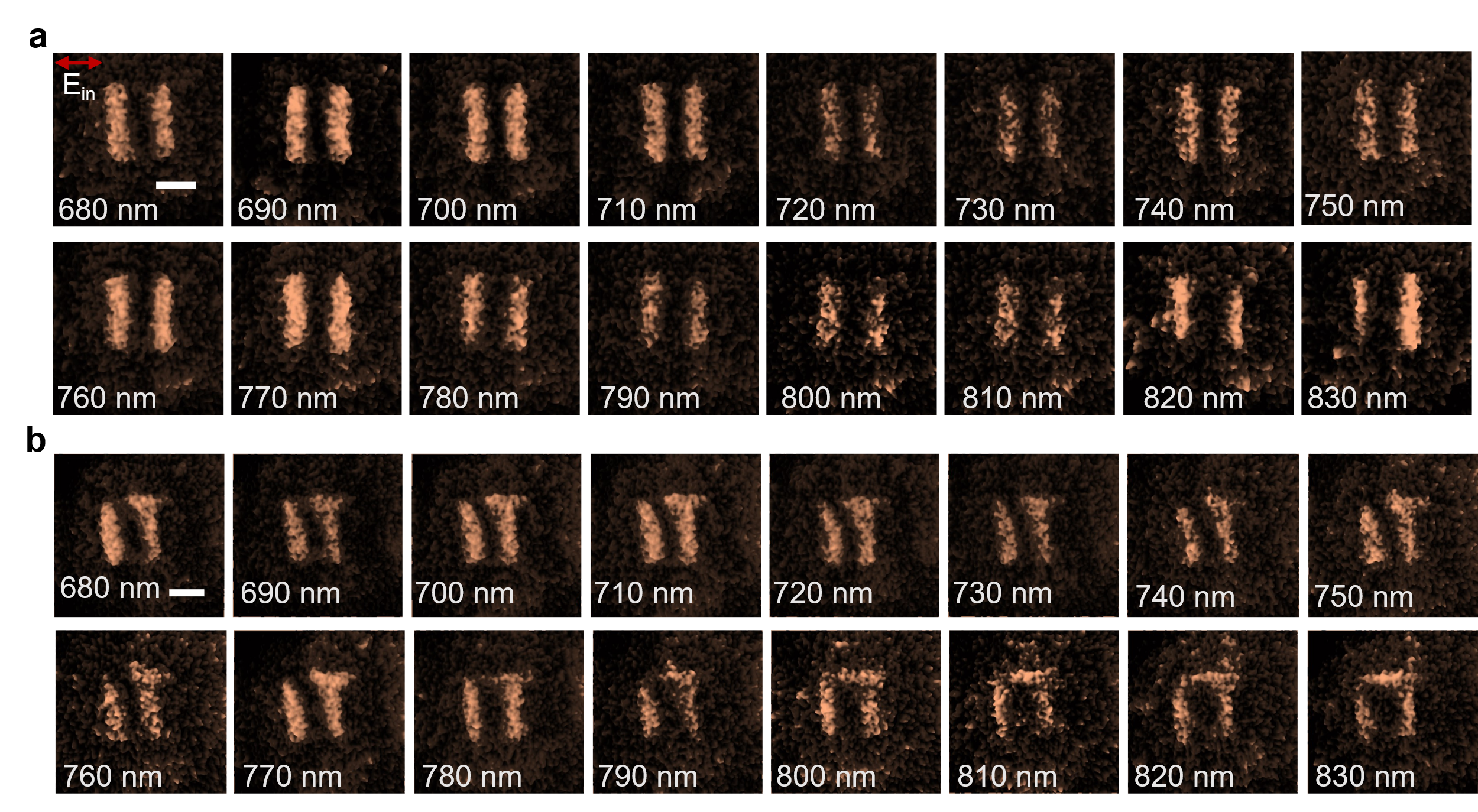
**

**Fig. S4** Measured near-field distributions of achiral (**a**) and chiral (**b**) meta-atoms obtained at time zero under horizontally polarized light excitation with different wavelengths (680-830 nm). Scale bar: 100 nm.


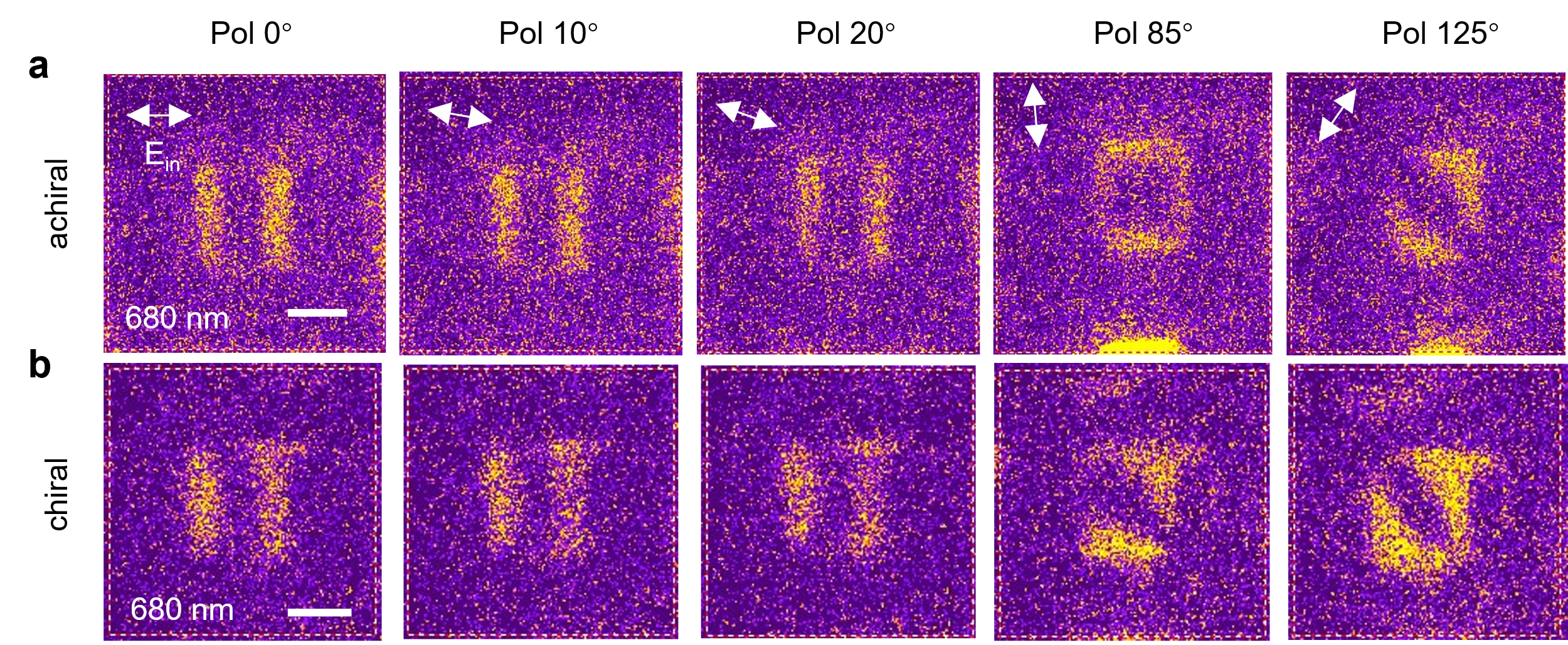


**Fig. S5** Near-field distributions of the achiral meta-atom (**a**) and chiral meta-atom (**b**) under 680 nm fs pulse excitation with polarization angles of 0°, 10°, 20°, 85°, and 125°. Scale bar: 100 nm.


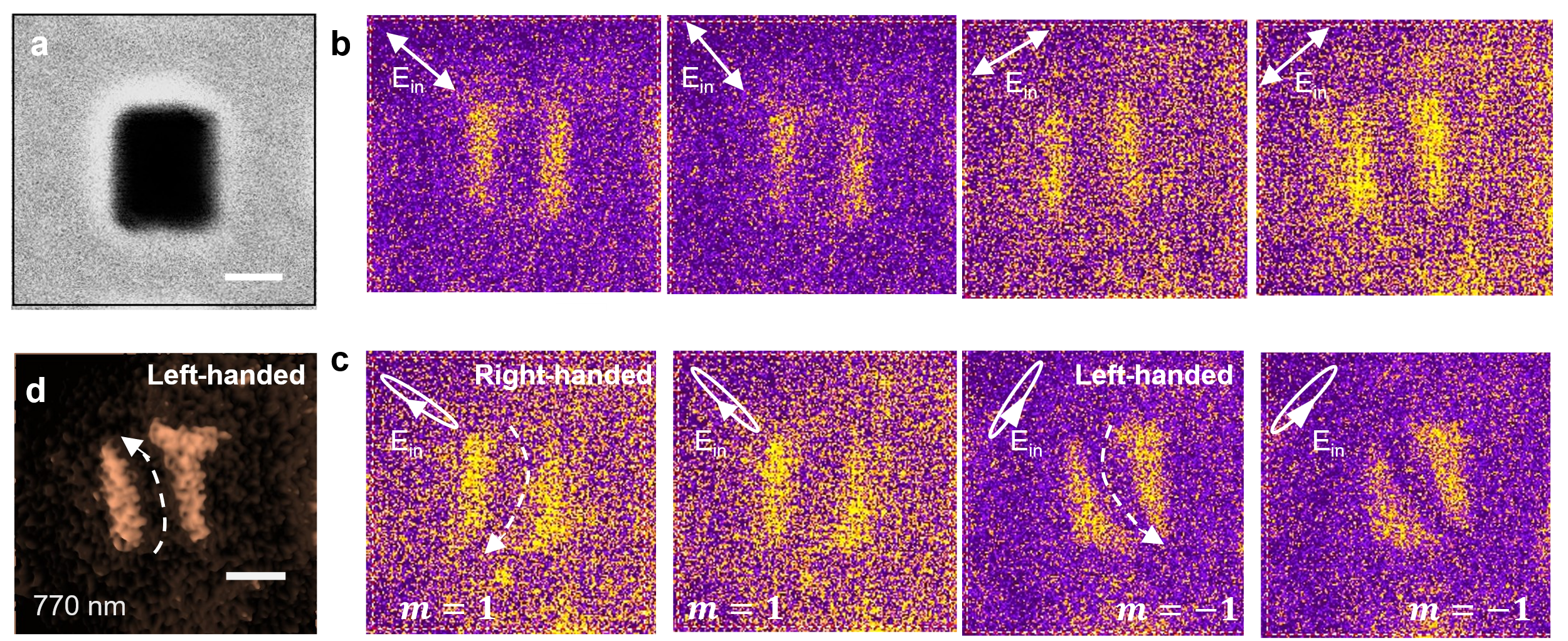


**Fig. S6 a**, SEM image of the achiral meta-atom. **b**, Near-field distributions of the achiral meta-atom under linearly polarized light excitation. **c**, Near-field distributions of the achiral meta-atom under right-handed elliptically polarized light excitation ($m = 1$) and left-handed elliptically polarized light excitation ($m = -1$). **d**, Near-field distribution of the chiral meta-atom under 770 nm fs pulse excitation with horizontally polarization. The dashed arrows indicate the distortion directions of the near-field distribution. Scale bar: 100 nm.

**Supplementary note 2. Definition and validation of the near-field ellipticity**

***Definition of the near-field ellipticity in achiral meta-atoms***

We propose a method to characterize the near-field optical response with an equivalent ellipticity parameter. To this end, we start from numerical simulations and analysis of the near-field distributions in an achiral square metasurface under light illumination with different ellipticity angles (*χ*) ranging from –90° to +90° (**Fig. S7**). The numerical simulations were performed using COMSOL Multiphysics. Given the achiral nature of the square structure, the near-field distribution is solely governed by the polarization state of the incident light. Simulation results indicate that the near-field profiles primarily arise from the coupling between the meta-atom and the projections of the incident polarization along the *x* and *y* axes. For instance, when $\chi=-90^{\circ}$, corresponding to linear polarization along the *y*-axis, the near-field is exclusively localized at the top and bottom edges of the structure. In contrast, under incident light with $\chi=-45^{\circ}$, which exhibits equal contributions from the *x*- and *y*-polarization components, the near-field distribution becomes symmetric with respect to both the *x*- and *y*-axes. Inspired by the definition of ellipticity for elliptically polarized light, i.e. $\varepsilon=B/A$ (*A* and *B* denote the major and minor axes of the elliptically polarized light, respectively), we define a near-field ellipticity for the meta-atoms as $\varepsilon^{'}=m\times I_{y}/I_{x}$. Here, $I_{x}$ and $I_{y}$ represent the integrated near-field intensity induced by the *x*- and *y*-projected components of the incident light, respectively; $m$ is a handedness factor, with $m=+1$ for clockwise near-field rotation and $m=-1$ for counterclockwise rotation.

**
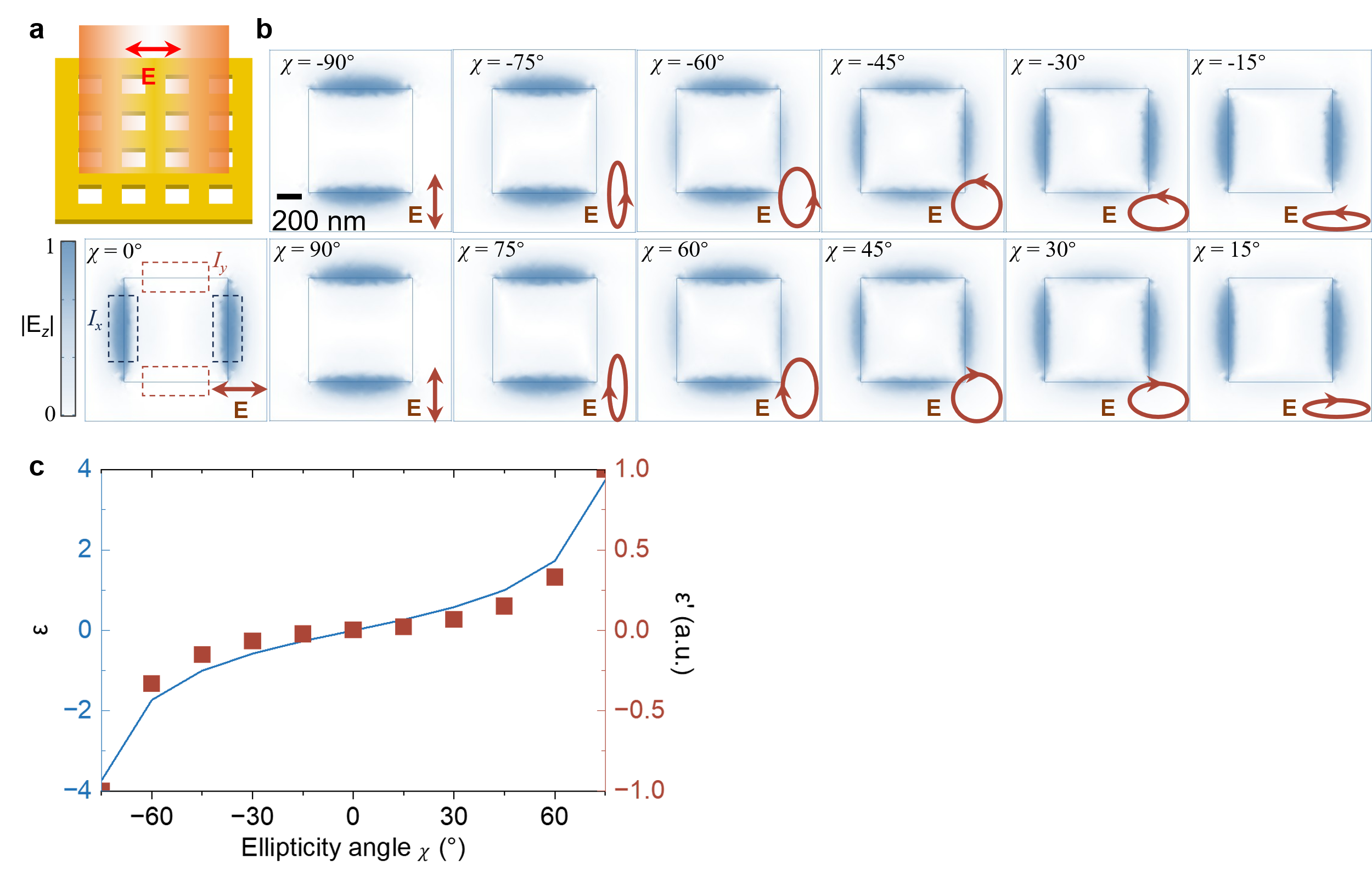
**

**Fig. S7 Near-field distributions of a square metasurface under incident light with different ellipticity angles. a**, Schematic of the square metasurface excited at 700 nm. **b**, Simulated electric field ($E_{z}$) distributions of the achiral meta-atom under light excitation with different ellipticity angles (-90°~ 90°). **c**, Comparison between near-field ellipticity $\varepsilon^{'}$ and incident light ellipticity $\varepsilon$.

***Extension the near-field ellipticity definition to chiral meta-atoms***

We generalize the definition to chiral structures by introducing the expression $\varepsilon=m\times I_{as}/I_{s}$, where $I_{as}$and $I_{s}$ denote the integrated near-field intensities of the near-field deviated from the incident light polarization and that along the incident light polarization, corresponding to asymmetric (*I_as_*) and symmetric (*I_s_*) near-field contributions (**Fig. S8**), respectively; $m$ characterizes the overall distribution rotation direction of the near-field, where $m = -1$ indicating a counterclockwise rotation and $m = 1$ representing a clockwise rotation. Finally, we applied this definition to a Γ-shaped metasurface consistent with that studied in our experiment. The calculated near-field ellipticity as a function of wavelength (**Fig. S8**) exhibits good agreement with experimental measurements, validating the proposed near-field ellipticity characterization approach.


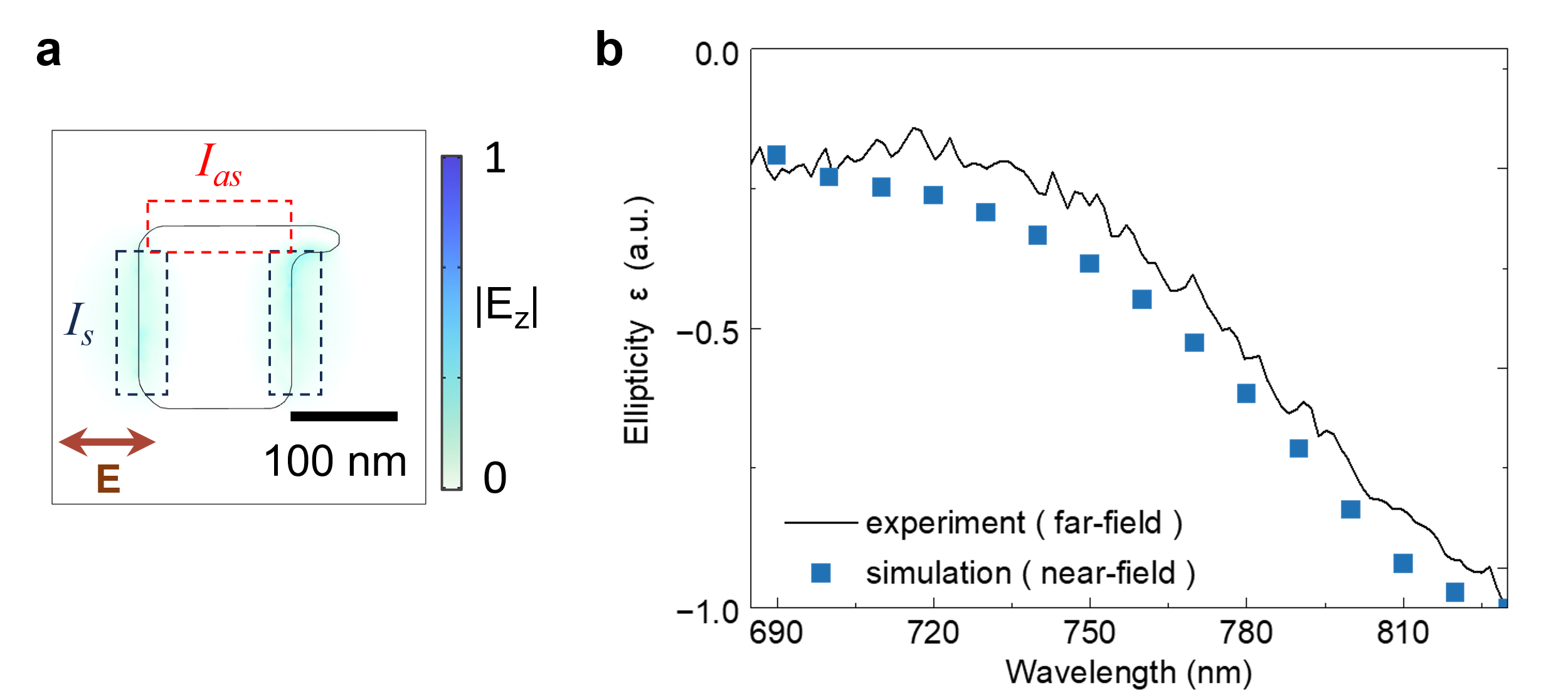


**Fig. S8 Wavelength dependence of the near-field ellipticity for the** **Γ-shaped metasurface.** **a**, Simulated electric field distribution at 700 nm. **b**, Comparison of the variation trends between the simulated near-field ellipticity and experimental far-field ellipticity for the Γ-shaped metasurface.


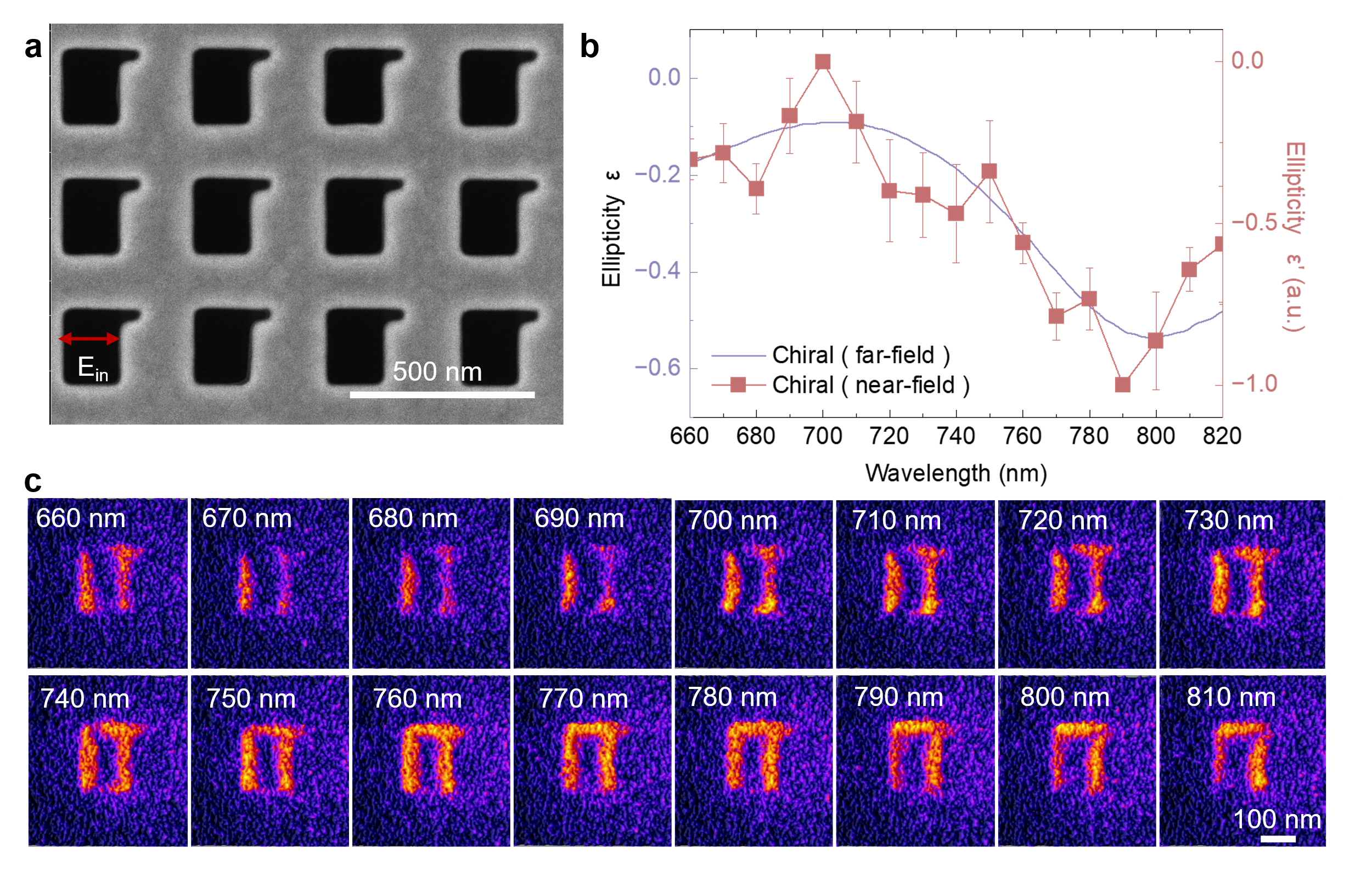


**Fig. S9 a**, SEM image of the chiral meta-atom with a 100-nm-thick gold film deposited on a 30-nm-thick Si_3_N_4_ substrate. **b**, Wavelength-dependent changes of far-field ellipticity angle $\varepsilon$and near-field ellipticity $\varepsilon^{'}$ of the transmitted light for the chiral metasurfaces under horizontally polarized light illumination (660-810 nm). Error bars represent standard deviations from 3 independent measurements. **c**, Measured near-field distributions of the chiral meta-atoms obtained at time zero under horizontally polarized light excitation with different wavelengths (660-810 nm).


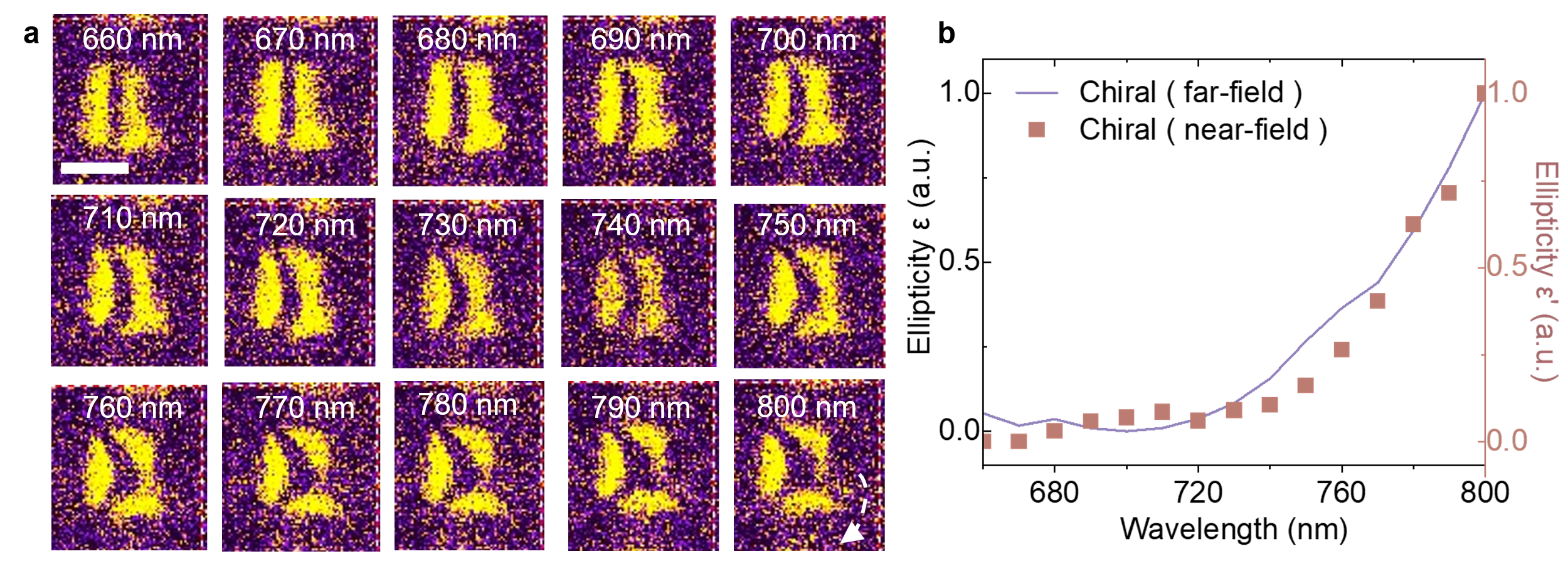


**Fig. S10** **a**, Measured near-field distributions of L-shape chiral meta-atoms (100-nm-thick Au film on a 30-nm-thick Si₃N₄ substrate) obtained at time zero under horizontally polarized light excitation with different wavelengths (660-800 nm). Scale bar: 200 nm. **b**, Wavelength-dependent changes of far-field ellipticity $\varepsilon$ and near-field ellipticity $\varepsilon^{'}$ of the transmitted light for the L-shape chiral metasurface under horizontally polarized light illumination (660-800 nm).

**
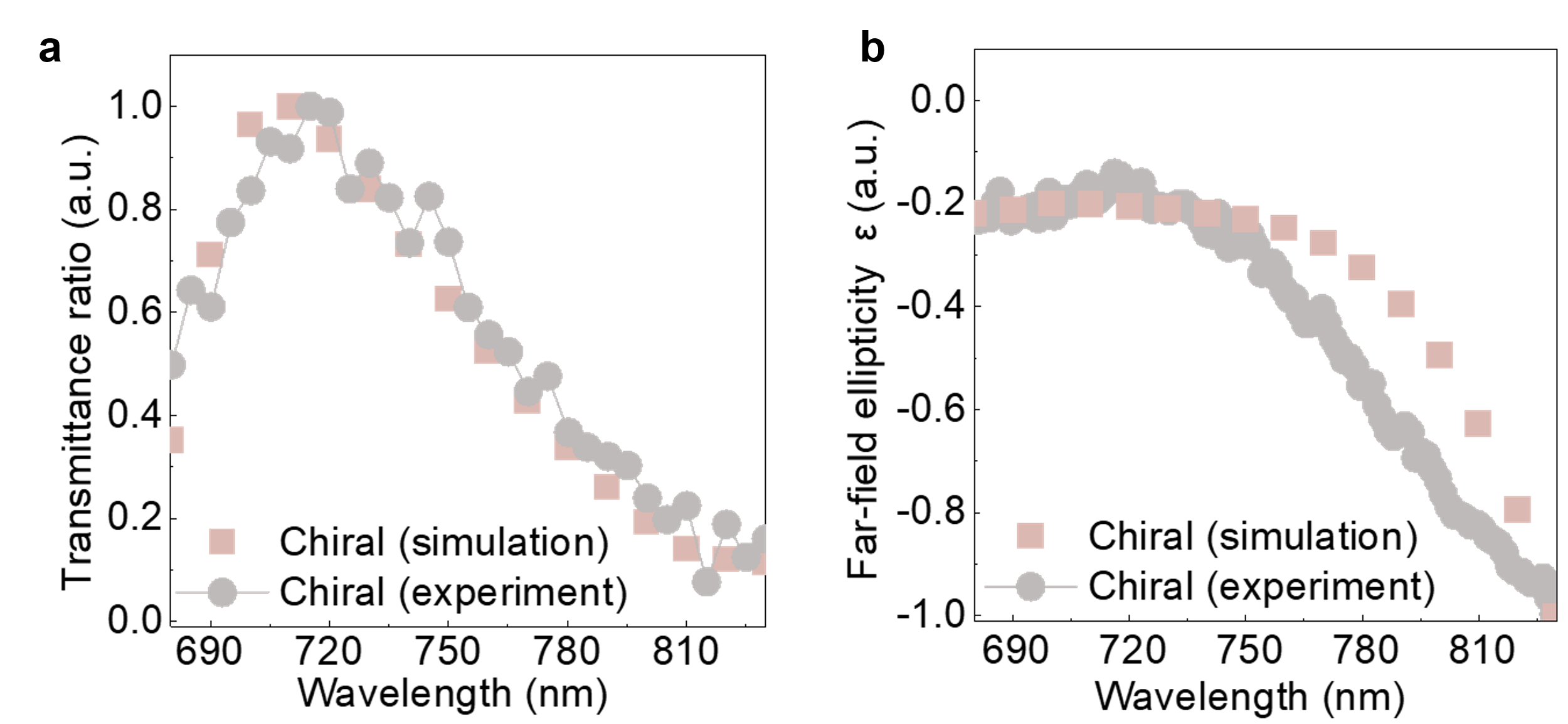
**

**Fig. S11** **a**, Wavelength-dependent transmittance ratio of the chiral metasurface. **b**, Far-field spectral variation of the ellipticity ($\varepsilon$) from 680 nm to 830 nm for the chiral metasurface.


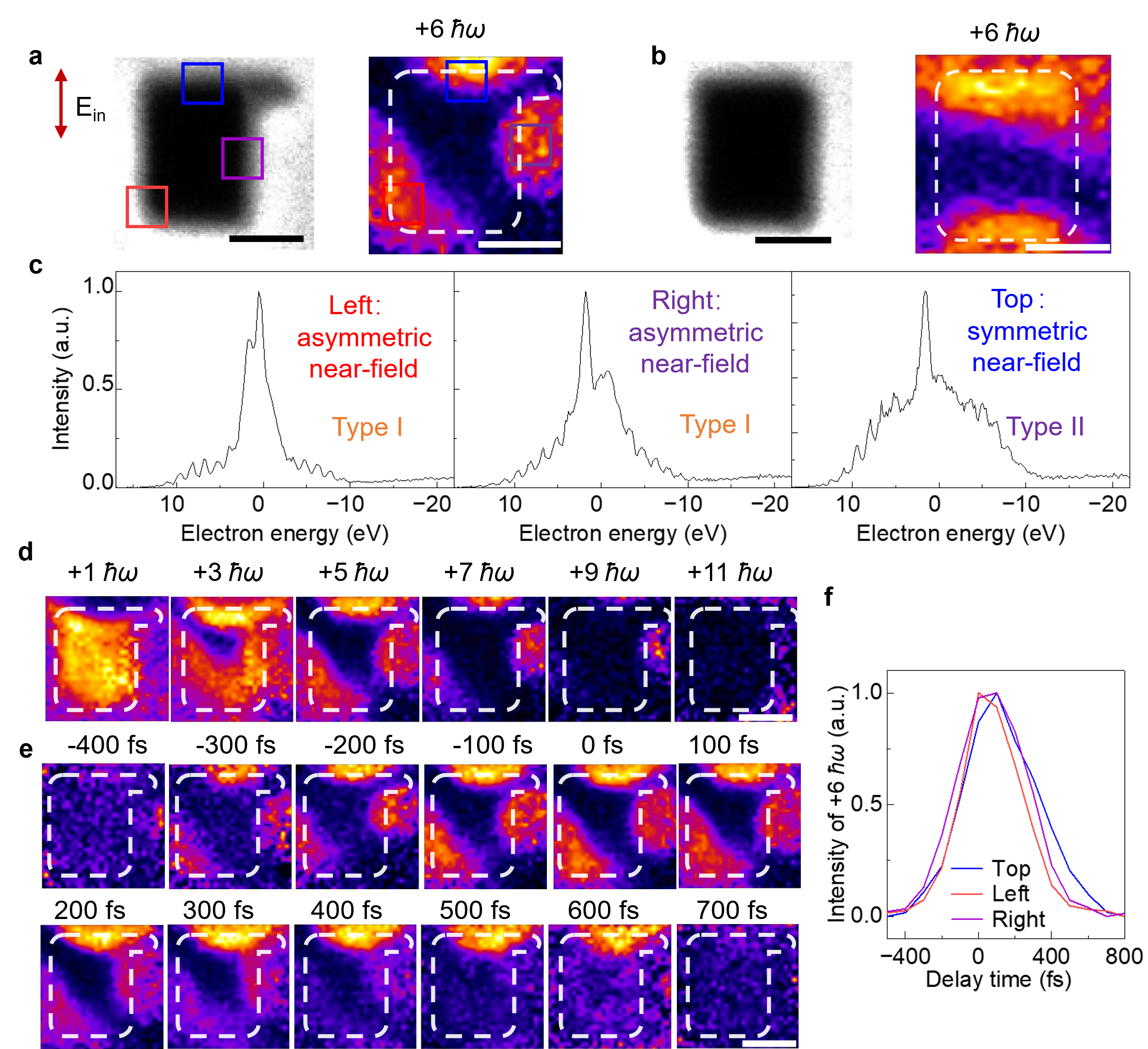


**Fig. S12** SEM images and energy gain images at +6 *ħω* of the chiral (**a**) and achiral (**b**) Au meta-atoms with an 80-nm-thick gold film deposited on a 30-nm-thick Si_3_N_4_ substrate under 830 nm vertically polarized laser excitation. **c**, Electron energy spectroscopy data showing multiple photon absorption and emission collected at different rectangular positions (blue rectangle: symmetric near-field, red and purple rectangles: asymmetric near-fields) indicated in **a**. **d**, Energy gain images at +1 *ħω* to +11 *ħω* obtained at time zero for the chiral meta-atom. **e**, Time-dependent energy gain images obtained at +6 *ħω* for the chiral meta-atom (see **c** for energy gain spectra). **f**, Time-dependent experimental probabilities of the electron-photon interaction intensity (+6 *ħω*) of the near-fields at different positions. Scale bar: 100 nm.

Under vertically polarized fs laser illumination at 830 nm, energy gain images at +6 *ħω* of the achiral meta-atom reveal a vertically symmetric near-field distribution (**Fig. S12b**). However, due to the effects of the geometric chirality of the structure, the energy gain image at +6 *ħω* exhibits asymmetric near-field features around the red (left) and purple (right) rectangles, which deviates from the original laser polarization direction (**Fig. S12a**). The optical phase modulation of the electrons through these two regions of the chiral meta-atom demonstrates that the transitions are predominantly governed by the sequential multilevel excitation (type I process in **Fig. 4b**). In contrast, the top region (blue rectangle) reveals a symmetric near-field distribution aligned with the original polarization, indicating that the electron transitions in this region are dominated by the multipath interference (type II process in **Fig. 4b**). **Fig. S12d** shows the energy gain images obtained at time zero for the chiral meta-atom by selecting electrons of different energy states (+1 *ħω*, +3 *ħω*, +5 *ħω*, ……, and +11 *ħω*). By selecting the electrons that gained photon energy of +6 *ħω*, we determined that the maximal interaction time points of the asymmetric near-fields at the red (left) and purple (right) rectangle areas are shifted by ~75 fs and ~25 fs, respectively, relative to the symmetric near-field at the blue (top) rectangle area (**Fig. S12f**). Notably, the asymmetric near-field which interacts with 6 photons dissipates faster than the symmetric near-field (**Fig. S12e**).

**Supplementary References**

1. Feist A, et al. Quantum coherent optical phase modulation in an ultrafast transmission electron microscope. *Nature* **521**, 200-203 (2015).

2. García de Abajo, F. J., Asenjo-Garcia, A. & Kociak, M. Multiphoton absorption and emission by interaction of swift electrons with evanescent light fields. *Nano Letters* **10**, 1859-1863 (2010).

3. Park ST, et al. Photon-induced near-field electron microscopy (PINEM): Theoretical and experimental. *New Journal of Physics* **12**, 123028 (2010).

4. Zheng D, et al. Nanoscale visualization of a photoinduced plasmonic near-field in a single nanowire by free electrons. *Nano Letters* **21**, 10238-10243 (2021).

5. Park S. T. & Zewail AH. Photon-induced near-field electron microscopy: Mathematical formulation of the relation between the experimental observables and the optically driven charge density of nanoparticles. *Physical Review A* **89**, 013851 (2014).

6. García de Abajo, F. J. Optical excitations in electron microscopy. *Reviews of Modern Physics* **82**, 209-275 (2010).
